# Potential Role of Inflammation-Promoting Biliary Microbiome in Primary Sclerosing Cholangitis and Cholangiocarcinoma

**DOI:** 10.3390/cancers14092120

**Published:** 2022-04-24

**Authors:** Katsuyuki Miyabe, Vinay Chandrasekhara, Nicha Wongjarupong, Jun Chen, Lu Yang, Stephen Johnson, Nicholas Chia, Marina Walther-Antonio, Janet Z. Yao, Sean C. Harrington, Cynthia K. Nordyke, John E. Eaton, Andrea A. Gossard, Sharad Oli, Hamdi A. Ali, Sravanthi Lavu, Nasra H. Giama, Fatima A. Hassan, Hawa M. Ali, Felicity T. Enders, Sumera I. Ilyas, Gregory J. Gores, Mark D. Topazian, Purna C. Kashyap, Lewis R. Roberts

**Affiliations:** 1 Division of Gastroenterology and Hepatology, Mayo Clinic, Rochester, MN 55905, USA; miyabe.katsuyuki@mayo.edu (K.M.); chandrasekhara.vinay@mayo.edu (V.C.); nwongjarupong@gmail.com (N.W.); nordyke.cynthia@mayo.edu (C.K.N.); eaton.john@mayo.edu (J.E.E.); gossard.andrea@mayo.edu (A.A.G.); drsharadoli@gmail.com (S.O.); nasroj@gmail.com (H.A.A.); sravanthilavu@gmail.com (S.L.); giama.nasra@mayo.edu (N.H.G.); hassa548@umn.edu (F.A.H.); ali.hawa@mayo.edu (H.M.A.); ilyas.sumera@mayo.edu (S.I.I.); gores.gregory@mayo.edu (G.J.G.); topazian.mark@mayo.edu (M.D.T.); kashyap.purna@mayo.edu (P.C.K.); 2 Department of Gastroenterology, Japanese Red Cross Aichi Medical Center Nagoya Daini Hospital, Nagoya 466-8650, Japan; 3 Division of Computational Biology, Mayo Clinic, Rochester, MN 55905, USA; chen.jun2@mayo.edu (J.C.); yang.lu@mayo.edu (L.Y.); johnson.stephen1@mayo.edu (S.J.); chia.nicholas@mayo.edu (N.C.); enders.felicity@mayo.edu (F.T.E.); 4 Microbiome Program, Center for Individualized Medicine, Mayo Clinic, Rochester, MN 55905, USA; waltherantonio.marina@mayo.edu (M.W.-A.); yao.zhengzhi@mayo.edu (J.Z.Y.); harrington.sean@mayo.edu (S.C.H.); 5 Department of Surgery, Mayo Clinic, Rochester, MN 55905, USA; 6 Department of Obstetrics & Gynecology, Mayo Clinic, Rochester, MN 55905, USA; 7 Department of Pathology, Westchester Medical Center, Valhalla, NY 10595, USA

**Keywords:** bile microbiome, cholangiocarcinoma, primary sclerosing cholangitis

## Abstract

**Simple Summary:**

This is the first study to investigate both bile and stool microbiota profiles in PSC and CCA, mostly perihilar CCA. Samples with less than 2000 reads were excluded from the compositional analysis to reduce false associations. We adjusted for demographic and clinical factors influencing the biliary and stool microbiota in PSC and CCA patients. Bile and stool have different profiles of microbiota, although the bile and stool microbiome from the same subject showed more similarity than those from different subjects. Increased species richness and abundance of Fusobacteria in bile was correlated with the duration of PSC and characterized the bile of CCA patients. The unique microbial signature in the bile of patients with CCA raises the possibility of a role for microbiota-driven inflammation in the pathogenesis of perihilar CCA.

**Abstract:**

Background: Primary sclerosing cholangitis (PSC) is a major risk factor for cholangiocarcinoma (CCA). We investigated biliary and fecal microbiota to determine whether specific microbes in the bile or stool are associated with PSC or CCA. Methods: Bile was obtained from 32 patients with PSC, 23 with CCA with PSC, 26 with CCA without PSC, and 17 controls. Over 90% of bile samples were from patients with perihilar CCA. Stool was obtained from 31 patients with PSC (11 were matched to bile), 16 with CCA with PSC (10 matched to bile), and 11 with CCA without PSC (6 matched to bile). Microbiota composition was assessed using 16SrRNA-marker-based sequencing and was compared between groups. Results: Bile has a unique microbiota distinguished from negative DNA controls and stool. Increased species richness and abundance of Fusobacteria correlated with duration of PSC and characterized the biliary microbiota in CCA. Stool microbiota composition showed no significant differences between groups. Conclusions: We identified a unique microbial signature in the bile of patients with increased duration of PSC or with CCA, suggesting a role for microbiota-driven inflammation in the pathogenesis and or progression to perihilar CCA. Further studies are needed to test this hypothesis.

## 1. Introduction

Primary sclerosing cholangitis (PSC) is a chronic, fibroinflammatory, cholestatic liver disease of unknown etiopathogenesis, which is a major risk factor for cholangiocarcinoma (CCA) [1,2,3]. CCA is a lethal biliary tract cancer with suboptimal treatment outcomes [4,5]. PSC and CCA cause chronic biliary obstruction that may lead to infectious cholangitis and liver dysfunction. Recently, the “PSC microbiota” hypothesis, which incorporates the observations that some patients with PSC have no detectable bowel disease and/or normal intestinal permeability [1] but may have an abnormal repertoire of microbial metabolites or an aberrant response to them, has been proposed [6,7]. The mechanisms by which microbial metabolites may contribute to CCA development are under active investigation.

Studies of the bile microbiome have described several phyla that are significantly increased in patients with distal CCA compared to patients with bile duct stones [8,9]. Furthermore, the abundances of several bacterial genera were different in stool microbiota of patients with intrahepatic CCA compared with patients with hepatocellular carcinoma or liver cirrhosis and in normal individuals [2]. However, no previous reports have focused on patients with perihilar CCA or compared the bile and stool microbiomes of patients with PSC or CCA.

We aimed to compare the biliary and stool microbiota, investigate the roles of their microbiota in PSC and CCA, and determine whether specific microbiota are associated with duration of PSC or progression to CCA.

## 2. Materials and Methods

### 2.1. Patient Selection, Sample Collection, and Data Collection

Patients were recruited through a Mayo Clinic Institutional Review Board (IRB) approved biorepository protocol, “An International Registry of Patients with or at Risk for Hepatobiliary Cancers, Including Hepatocellular Carcinoma, Cholangiocarcinoma, and Gallbladder Adenocarcinoma” (IRB# 707-03). After written informed consent, patients undergoing Endoscopic Retrograde Cholangiopancreatography (ERCP) at Mayo Clinic Rochester for known or suspected PSC and/or CCA were enrolled in the study. The use of bile and stool samples collected under IRB#707-03 for the present study was approved by the Mayo Clinic IRB (IRB# 16-004650). Diagnostic criteria for PSC included an increased serum alkaline phosphatase level that persisted for more than 6 months, typical cholangiographic findings of bile-duct strictures detected by means of either magnetic resonance cholangiopancreatography (MRCP) or ERCP, and exclusion of causes of secondary sclerosing cholangitis [10]. The diagnosis of CCA was confirmed by the pathologic findings obtained from the bile duct endoscopically or percutaneously, or a progressive clinical course with radiographic images consistent with malignancy, after exclusion of benign biliary strictures.

Bile samples were collected during endoscopic or surgical procedures and were collected twice if possible. Stool samples were collected on site at Mayo Clinic or at the patient’s home and mailed back to Mayo Clinic. For controls, patients with cholelithiasis and/or choledocholithiasis undergoing ERCP or surgery were consented.

Data on gender, age, BMI, race, current smoking status, current alcohol intake, current or past illness (particularly, inflammatory bowel disease, irritable bowel syndrome, autoimmune diseases, and cancers), current or past treatment (antibiotic use within the past week, steroid use, immunosuppressant use, anti-cancer treatment such as chemotherapy and radiotherapy, supplement use, gastrointestinal surgery, and placement of plastic or metallic biliary stents), and the presence of biliary strictures at the time of initial sample collection were obtained or abstracted from the medical record. The status of liver fibrosis in PSC/CCA patients was assessed at baseline using MR elastography. For CCA patients, tumor stage according to TNM classification and history of chemotherapy and/or radiotherapy were collected.

### 2.2. Microbiome Analyses

Genomic DNA extraction, next-generation sequencing, and bioinformatics processing of the sequenced reads are fully described in the Supplementary Materials. Both bile and stool 16S rDNA amplicons were sequenced on one lane of a MiSeq (Illumina Inc., San Diego, CA, USA). Pre-processed sequence files were then analyzed via the hybrid de novo bioinformatics pipeline [11] with the default parameter settings to obtain the operational taxonomic unit (OTU) table. OTUs were assigned taxonomy using the Ribosomal Database Project (RDP) classifier trained on the Greengenes database (v13.5) and a phylogenetic tree was built based on FastTree [12]. Due to their low bacterial content and the inability to reliably characterize their bacterial profiles and exclude the influence of environmental contamination, samples with fewer than 2000 reads were excluded from the analyses of the microbiome profiles at QC. After QC of the study samples, 3,751,425 reads remained (median: 23,652 reads per sample, range: 2220 to 58,443 reads per sample, lower and upper quartiles: 16,853 and 30,939 reads per sample). The OTUs belong to 18 phyla, 98 families and 162 genera based on the RDP classifier on the Greengenes database.

### 2.3. Statistical Analyses

Statistical procedures are fully described in the Supplementary Materials. Differences in patient characteristics among disease groups were compared using the Chi-square test or Fisher’s exact test for categorical variables and the Kruskal–Wallis test for continuous variables. For the microbiome analyses, sources of variability in the dataset were identified by testing associations between the overall microbiota composition and various clinical variables using the PERMANOVA-based omnibus test [13]. Detailed comparative analyses of the bile and stool microbiota from controls vs. PSC, PSC vs. CCA with PSC (CCA w PSC), and controls vs. CCA without PSC (CCA wo PSC), were performed at α-diversity, β-diversity and taxa abundances. To test whether the bile and stool samples from the same subject were similar, a distance-based permutation approach was used. Statistical analyses were performed in R 3.3.2 and JMP software (version 10.0.2; SAS Institute, Cary, NC, USA).

## 3. Results

### 3.1. Sample Collection and Patient Characteristics

**Figure 1:**
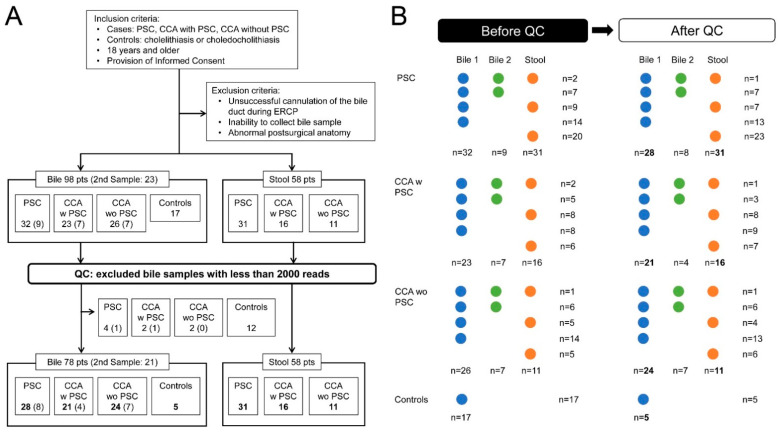
(**A**) Flow chart of the study with quality control after sample collection. (**B**) Outline of sample collection. PSC, primary sclerosing cholangitis; CCA, cholangiocarcinoma; QC, quality control; CCA w PSC, CCA with PSC; CCA wo PSC, CCA without PSC.

Bile samples, designated Bile 1, were collected at ERCP or during cholecystectomy from three groups of patients, PSC without CCA (PSC, 32 patients), CCA w PSC (23 patients), sporadic CCA wo PSC (26 patients), and controls with cholelithiasis or choledocholithiasis (17 patients). In a subset of patients, a second sample, designated Bile 2, was obtained at the time of a subsequent ERCP, including PSC (nine patients), CCA w PSC (seven patients), and CCA wo PSC (seven patients). Of the 58 eligible patients with stool samples, 31 (53%) were from PSC, 16 (28%) from CCA w PSC, and 11 (19%) from CCA wo PSC. (Figure 1A,B).

In the bile samples, there were significant differences between groups in the age distribution, BMI, presence of coexisting cholelithiasis, choledocholithiasis, or ileal pouch, the rates of blood leukocytosis, current smoking status, presence of inflammatory bowel disease (IBD), or hypertension, and treatment with chemotherapy, radiotherapy, or stent placement. (Table S1).

For quality control (QC) of bacterial DNA, samples with fewer than 2000 reads were excluded from the analyses of the microbiome profiles (*n* = 22, all bile samples). The 22 bile samples with low reads were from five PSC (four bile samples from the first collection and one bile sample from the second collection in a different patient), three CCA w PSC (two bile samples from the first collection and one bile sample from the second collection in a different patient), two CCA wo PSC from the first collection, and 12 patients in the bile control group (Figure 1A,B). No stool samples were excluded. In 29 cases, stool samples were collected from patients in whom bile could not be obtained at ERCP. The final data set contained 99 bile samples, including 78 samples collected at the first ERCP from 77 patients, or at surgery for one control patient, and 21 samples collected at the second ERCP from 21 patients. All 58 stool samples from 58 subjects were included (Figure 1A,B).

Of the 78 bile samples collected at the first ERCP, 28 (36%) were from PSC, 21 (27%) from CCA w PSC, 24 (31%) from CCA wo PSC, and 5 (6%) from control patients. Of these patients, 22 (28%) were women, 74 (95%) were white, 21 (27%) were age >65 years old, 19 (24%) had BMI > 30 kg/m^2^, 42 (54%) were alcohol users, and 27 (35%) were smokers. Of the patients from whom first ERCP bile samples were obtained, blood leukocytosis suggestive of potential cholangitis was observed in 2 of 28 (7%) PSC, 6 of 20 (29%) CCA w PSC, 4 of 24 (17%) CCA wo PSC, and 0 of 6 (0%) control patients. Of the Bile 1, blood leukocytosis suggestive of potential cholangitis was observed in 2 of 28 (7%) PSC, 6 of 20 (29%) CCA w PSC, 4 of 24 (17%) CCA wo PSC, and 0 of 6 (0%) control patients. Of the Bile 2 obtained from 19 patients, one of four (25%) CCA w PSC had blood leukocytosis. (Table 1).

Thirty-six (46%) bile samples from 20 PSC and 16 CCA w PSC were obtained from patients with coexisting IBD. Antibiotics had been taken within one week before sample collection by 27 (35%) patients from whom bile samples were obtained, including 10 PSC, 9 CCA w PSC, and 8 CCA wo PSC. Of the patients on antibiotics, nine (16%) of those providing bile samples took antibiotics continuously for more than a month. After QC, none of the patients from whom control bile samples were obtained used antibiotics before the bile collection. Antibiotics used included amoxicillin, fluoroquinolones, metronidazole, rifamycin, or vancomycin. The exact duration of antibiotic use in individuals on long-term antibiotic therapy could not be ascertained.

After QC, there remained significant differences between groups in the age distribution, presence of coexisting cholelithiasis or choledocholithiasis, IBD, and treatment with chemotherapy, radiotherapy, or stent placement in participants providing both bile and stool samples. In participants providing stool samples, the rate of blood leukocytosis was also significantly different between groups (Table 1 and Table 2).

### 3.2. Bile Has a Unique Microbiota Distinguished from the Negative DNA Controls

**Figure 2:**
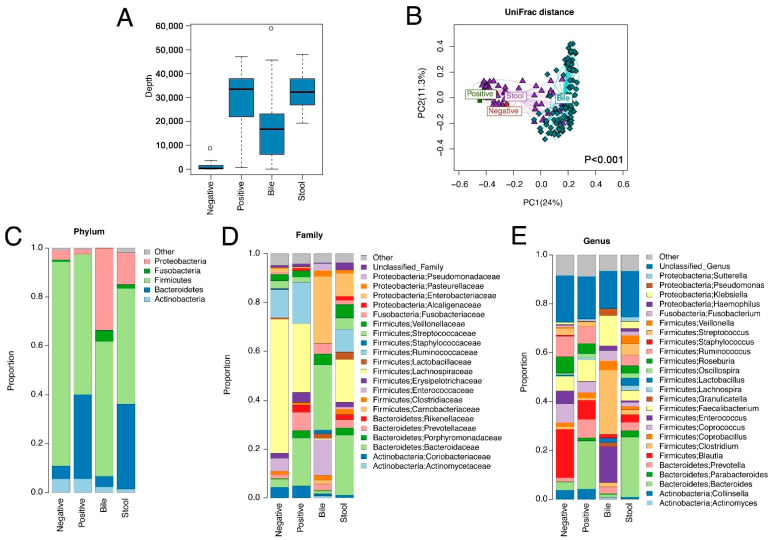
Microbiome variation across bile, stool, negative and positive controls. The negative controls were empty tubes processed alongside the DNA extractions for the samples, and positive controls were derived from stool samples pooled from 20 healthy subjects. (**A**) Sequence depth distribution shows that bile samples have a higher sequence depth than negative controls. (**B**) Ordination plot based on unweighted UniFrac distance reveals a distinct microbiota structure for bile samples. The statistical significance is confirmed by PERMANOVA (*p* < 0.001, bile vs. other sample types). (**C**–**E**) Average microbiota profiles in each sample type show that bile has a unique microbiota profile different from negative controls.

**Figure 3:**
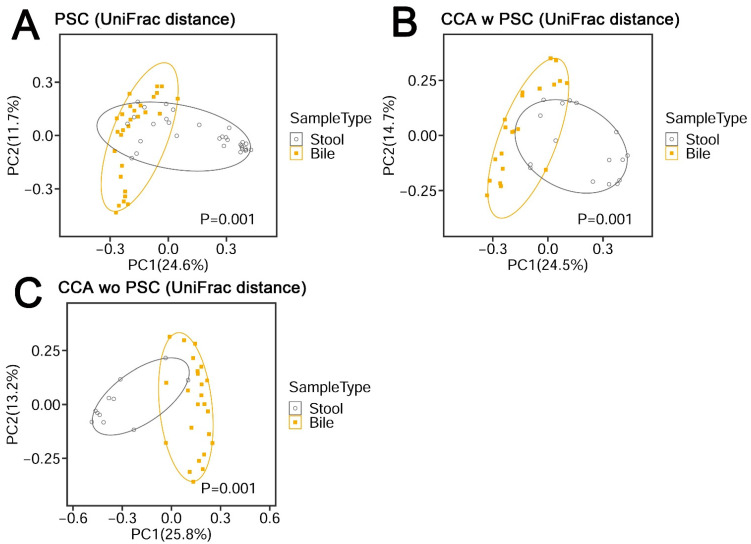
β-diversity metrics of PSC (**A**), CCA w PSC (**B**), and CCA wo PSC (**C**) samples. UniFrac distance consists of unweighted UniFrac distance.

Bile samples had significantly lower sequencing depths than stool samples (Figure 2A), reflecting their lower bacterial content. The bile samples had much higher sequencing depths than negative DNA controls (Figure 2A) and had a different microbiota profile than stool samples, showing differences in β-diversity (Figure 2B and Figure 3A–C; PERMANOVA omnibus test, *p* < 0.001), indicating both that contamination from stool was not a serious issue and that bile has a unique microbiota.

In the study cohort, the most common phylum, family, and genus in bile samples were Firmicutes, Enterobacteriaceae, and Streptococcus, respectively, whereas the most common phylum, family, and genus in stool samples were Firmicutes, Bacteroidaceae, and Bacteroides, respectively (Figure 2C–E).

**Figure 4:**
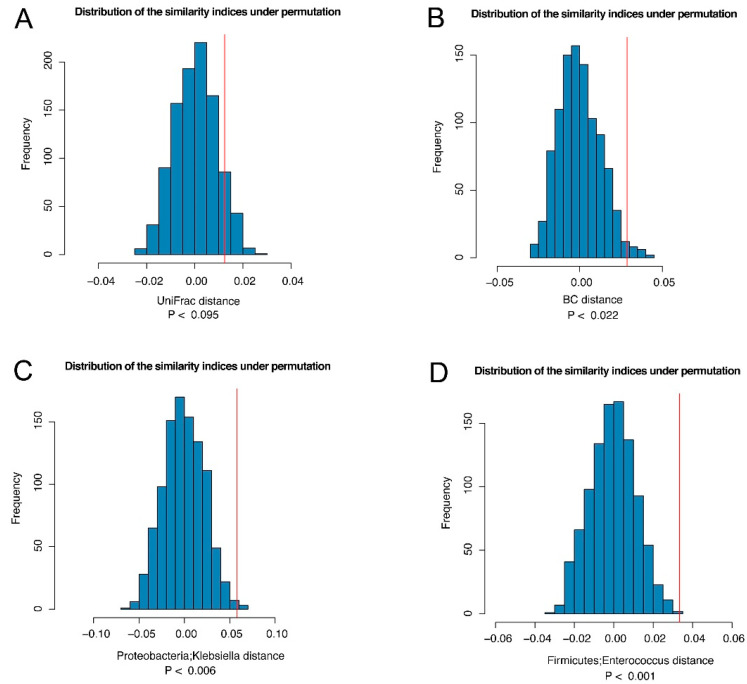
The similarity between the bile and stool microbiome from the same subject. We compared the average distance between the bile and stool samples from the same subjects (Dw) to the average distance between the bile and stool samples from different subjects (Db) based on UniFrac distance (**A**) and Bray–Curtis distance (**B**). If Db > Dw, it indicates that the bile and stool from the same subject is correlated. Thus, the test statistic Ts = (Db–Dw) can be interpreted as the similarity index. To establish significance, the observed similarity index (red vertical line) was compared to that under permutation (no correlation) to establish statistical significance. Two genera of Klebsiella (**C**) and Enterococcus (**D**) were identified to drive the “similarity” at FDR < 20%.

Bile and stool samples showed different microbiota profiles. Twenty-five subjects had both bile and stool samples collected, allowing us to assess the similarity of the microbiota of the bile and stool samples from the same subject. We used the distance-based permutation approach described in the Methods section. Based on the UniFrac and Bray-Curtis distance, we observed *p* values of 0.095 and 0.022, respectively, indicating that the microbiota of the bile and stool samples from the same subjects were correlated (Figure 4A,B) and that similar microbiota spectra may reflect specific disease development in both the bile ducts and intestinal tract.

Conversely, to identify taxa that were more similar from the same subject, we defined a taxon-specific distance for each taxon (Supplementary Materials) and repeated the same permutation test. At a false discovery rate (FDR) of 20%, we identified two genera: Proteobacteria Klebsiella and Firmicutes Enterococcus, which were more similar between the bile and stool samples from the same subject (Figure 4C,D).

### 3.3. Demographic and Clinical Factors Influencing the Biliary and Stool Microbiota in PSC and CCA Patients

According to the PERMANOVA test results, several clinical variables were associated with the composition of the bile and stool microbiota, particularly for the stool microbiota (Table 3). For bile samples, significant confounders were gender, antibiotic use, presence of an ileal pouch, and stent placement (Figure S1A–D), whereas for stool samples, significant confounders were antibiotic use, inflammatory bowel disease (IBD), presence of an ileal pouch, and presence of an ileostomy (Figure S2A–D). The association analyses adjusted for these significant variables (raw *p* < 0.05).

### 3.4. The Duration of PSC Is Correlated with Increased Species Richness and Abundance of Fusobacteria

**Figure 5:**
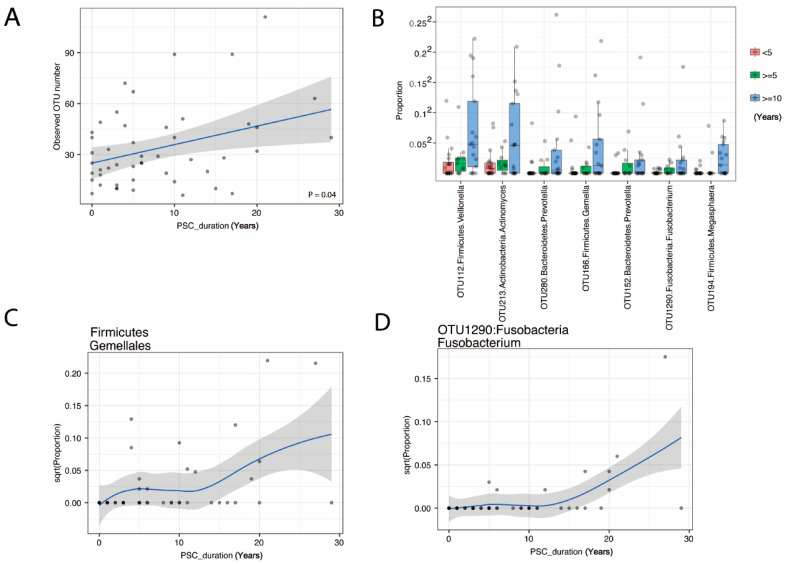
Changes in bile microbiome with PSC duration in years. Scatter plot of OTU number (**A**) shows an increase over PSC duration in years. Potential pathogens from 7 OTU (**B**) and the Order Firmicute; Gemellales (**C**) increase with PSC duration (FDR < 20%). An example detail from OTU1290:Fusobacteria Fusobacterium is shown in (**D**).

There were no statistically significant differences overall between PSC and control bile samples. However, when we investigated the correlation of species richness in bile with the duration of PSC, species richness was correlated with the duration of PSC (*p* = 0.05, Figure 5A). Moreover, we identified the order Firmicutes; Gemellales and seven OTUs belonging to Firmicutes, Actinobacteria, Bacteroidetes, and Fusobacteria as increasing with PSC duration (Table S2 and Figure 5B–D). Regarding Fusobacterium OTU1290 (Fusobacterium unclassified by RDP classifier), which was significantly associated with PSC duration, we performed a BLAST of the DNA sequence of OTU1290 against the NCBI bacterial DNA sequence database and found that the sequence has 100% match to the 16S rRNA gene of Fusobacterium nucleatum, canifelinum and hwasookii. Due to the resolution of 16S data, however, we were not able to resolve to the species/strain level; thus, future shotgun metagenomic sequencing will be needed to achieve a high-resolution view of the microbiota.

We performed correlation analysis between the Fusobacterium level and IBD, choledocholithiasis, and obesity. However, statistical significance was not reached for these variables (IBD: *p* = 0.25; choledocholithiasis: *p* = 0.88, obesity: *p* = 1.0. Wilcoxon rank sum test). We also performed predictive functional analysis using PICRUSt. No KEGG pathway was found to be associated with the level of Fusobacterium at 20% FDR.

### 3.5. The CCA Biliary Microbiota Is also Characterized by Increased Species Richness and Abundance of Fusobacteria

**Figure 6:**
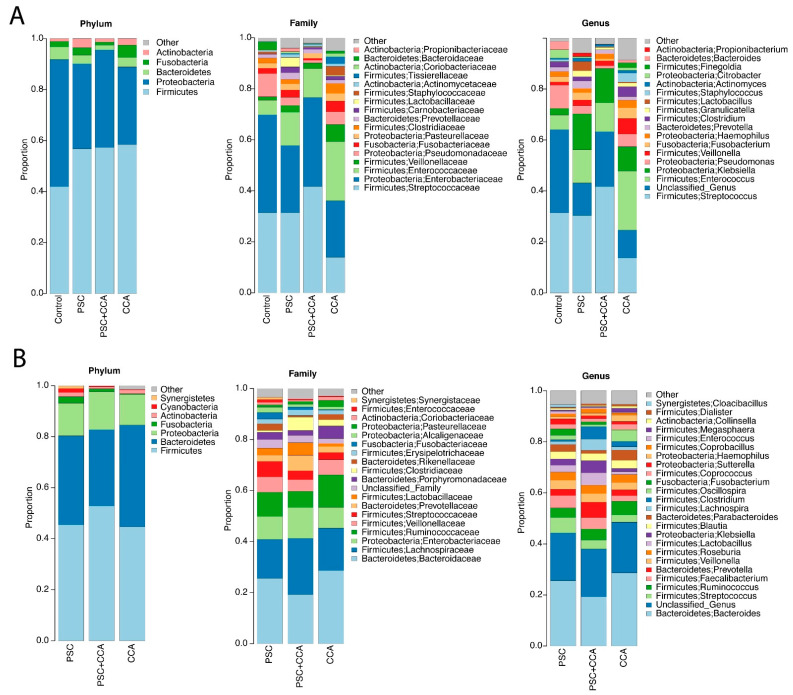
Average bile (**A**) and stool (**B**) microbiome profiles in PSC, CCA w PSC, and CCA wo PSC.

**Figure 7:**
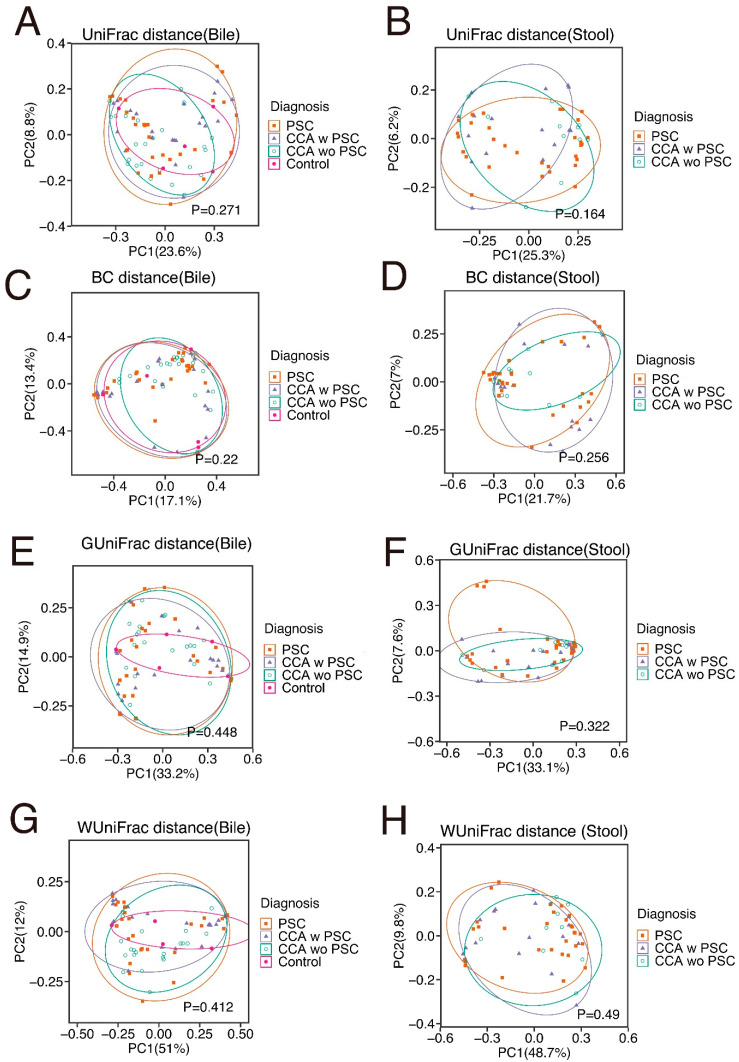
β-diversity metrics of all groups in bile (**A**,**C**,**E**,**G**) and stool (**B**,**D**,**F**,**H**) samples. UniFrac distance consists with unweighed UniFrac distance. BC, Bray–Cutis; GUniFrac, generalized UniFrac; WUniFrac, weighted UniFrac.

**Figure 8:**
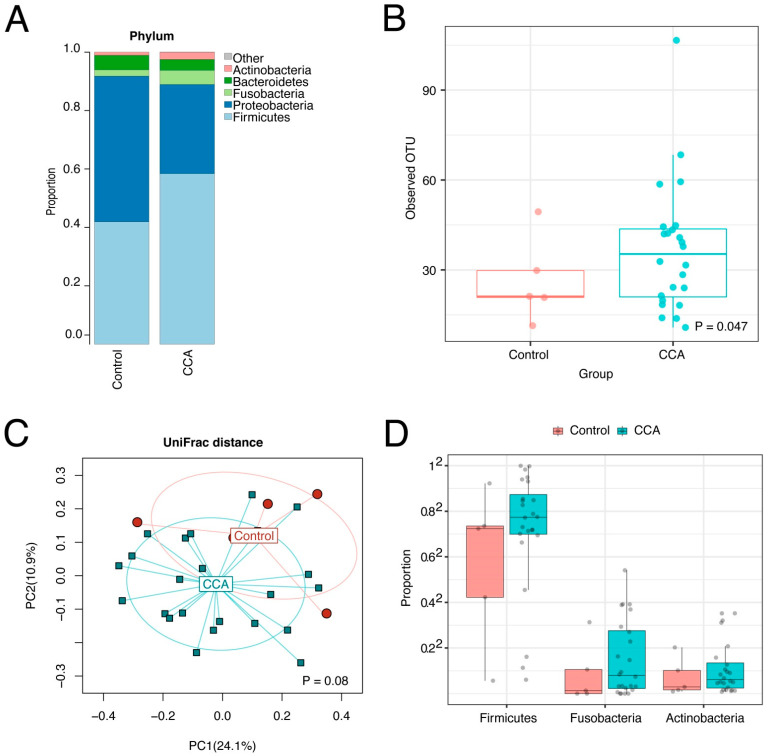
Bile microbiome profiles of CCA and controls. The bile microbiome profiles (**A**), the boxplots (**B**), and ordination plot analyzed by unweighted UniFrac distance (**C**) of CCA and controls shows species richness in CCA bile samples compared to controls. The bile microbiome boxplots (**D**) show increases in Firmicutes, Fusobacteria, and Actinobacteria in bile from CCA patients.

Entire microbiome profiles based on disease classification are shown in Figure 6A,B, and S3. β-diversity metrics of all groups in bile and stool samples are shown in Figure 7. The comparative analyses were performed after the adjustment of the significant covariates identified in Table 3. CCA bile samples had a significant difference in species richness (observed number of OTUs) from control bile samples (Figure 8A–C). Furthermore, CCA bile was characterized by increased abundance of Firmicutes, Fusobacteria, and Actinobacteria (FDR < 20%), compared to control bile samples (Figure 8D). It is notable that both Fusobacteria and Firmicutes Gemellales were also increased with increasing PSC duration in bile from patients with PSC.

### 3.6. The Stool Microbiota Was Not Statistically Different between PSC, CCA w PSC, or CCA wo PSC

We next compared the stool microbiota between PSC, CCA w PSC and CCA wo PSC based on α-diversity, β-diversity and taxa abundances. All the comparisons were adjusted for those potential confounders detected in Table 3. α-diversity measures were not significantly different between PSC and CCA w PSC, or between CCA w PSC and CCA wo PSC (linear regression *p* > 0.1 for both observed OTUs and Shannon index, see Table S3). β-diversity analysis did not reveal a significant difference in overall microbiota compositions between PSC and CCA w PSC, or between CCA w PSC and CCA wo PSC (PERMANOVA test *p* > 0.1 for all β-diversity measures, see Table S3). Differential abundance analysis at phylum, family, and genus level did not identify significantly differential taxa at 20% FDR.

## 4. Discussion

Our primary hypothesis was that the composition of the biliary microbiome is altered in patients with chronic biliary tract disease, such as is found in PSC, and that enhanced exposure to an altered microbiome may play a role in the progression of PSC and in CCA oncogenesis. Our study demonstrated that bile had a significant sequence depth with a composition that was different from stool microbiota and that a number of clinical variables affected the bile microbiome and the stool microbiome. As we hypothesized, bile and stool have different profiles of microbiota, although the bile and stool microbiome from the same subject showed more similarity than those from different subjects. More importantly, the duration of PSC affected the diversity of the bile microbiome, and dysbiosis of the bile microbiome also occurred in CCA. We could not design a comparison between IBD alone and IBD with PSC patients using bile samples, because it is not clinically indicated to perform ERCPs in patients with IBD without PSC. However, it has also been reported that Escherichia, Lachnospiraceae and Megasphera are increased in the stool of patients with PSC with IBD compared with healthy controls [14]. The authors concluded that the stool microbiome of patients with PSC with IBD was distinct from that of patients with IBD without PSC [15].

Several reports have described the bile microbiome [8,9,16,17,18,19]. Two reports compared the biliary microbiome between patients with bile duct stones and distal CCA, but no studies have compared the microbiome between bile and stool. Eighteen percent of the bile samples in our study, mostly control bile samples without PSC or CCA, had fewer than 2000 microbiome DNA reads. Bacteria DNA quantity and quality obtained from bile ducts might be low and therefore susceptible to environmental contamination in assays such as qPCR and amplicon sequencing-based microbial composition analyses [20]. Therefore, we excluded samples with low read numbers from the analyses in order to provide an adequate representation of the microbiome diversity, thus reducing bias and confounding and hopefully identifying true differences. The remaining 82% of bile samples had sufficient reads for microbiome DNA analysis, even when they were collected at the first attempt at ERCP before any opportunity for contamination by instrumentation, showing that bile has a measurable and specific microbiome in nature. A previous study detected positive bile cultures in only 38% of 956 bile samples, although the investigators only performed bile culture without microbiome DNA sequencing. In our study, only 5 out of 17 (29%) control bile samples qualified as having a measurable microbiome. None of the five control patients with a measurable microbiome had clinical evidence of bacterial infection and none were on antibiotic therapy. White blood cell counts, a surrogate marker of enteral bacterial contamination due to acute cholangitis or acute enteritis, were not significantly different between groups.

The duration of PSC affected the diversity of the bile microbiome, although PSC was characterized by decreased microbiota diversity in stool samples in a previous study [21]. This may be because bile originally has a relatively small number of bacteria, albeit it is not sterile in healthy subjects [19], and disease or other interventions may increase the bile microbiome, which is a contradictory dynamic compared to the stool microbiome. While dysbiosis of the stool microbiome may contribute to PSC and CCA pathogenesis [16,22], the role of bile microbiota in PSC and CCA development may be different. Furthermore, it is difficult to accurately assess the frequency of bacterial infections in PSC and CCA patients because many patients have low grade chronic cholangitis. Clinicians can usually only diagnose acute cholangitis when patients develop fever, jaundice, or abdominal pain. We therefore presumed that the duration of PSC or CCA better reflects the exposure to inflammation. A retrospective study of 399 PSC with IBD patients found that prolonged duration of IBD was associated with an increased risk of CCA [23], which is potentially analogous to our finding that increased species richness and abundance of Fusobacteria were correlated with the duration of PSC. In a previous study, PSC patients displayed ecological alterations of ductal bile [9], and Streptococcus abundance was also positively correlated with an increase in disease severity [9]. A previous study shows reduced biodiversity and increased abundances of Enterococcus faecalis in the bile of PSC patients compared with control bile samples obtained from patients with sporadic choledocholithiasis or patients with papillary adenoma [19], which is different from our findings. The study also found that microbial dysbiosis in PSC is associated with an increase in the proinflammatory and potentially cancerogenic bile acid taurolithocholic acid in bile [19]. PSC duration, which increases the microbial burden, is associated with CCA development, and the observed increase in Fusobacterium and Gemella, which are bacteria well known to cause inflammation [24,25,26], may be relevant to this process.

Fusobacteria were increased during the long-term course of PSC. Fusobacteria are common obligately anaerobic Gram-negative bacteria of the oral cavity that may act as a bridge between early and late colonizing bacteria in dental plaque and have a role in oral and extra-oral infections [27]. Increasing relative abundance of Fusobacteria coincides with increases in inflammatory markers such as IL-6, TNF-α, and IL-1β [28], which also suggests that increases in the relative abundance of Fusobacteria may induce local inflammation. An increase in Fusobacteria has also been previously associated with liver cirrhosis [29], IBD [30,31] and colorectal cancer [32]. Some Fusobacterium species are well known to produce biofilm in the oral cavity and cause complex infections or antibiotic resistance [16]. Our study has found that Fusobacterium nucleatum reported in papers [24,30] was statistically associated with PSC duration. However, in our study, statistical significance was not reached for IBD, obesity or choledocholithiasis according to our correlation analysis between the Fusobacterium level and these variables. We also performed predictive functional analysis of the microbiome data using Phylogenetic Investigation of Communities by Reconstruction of Unobserved States (PICRUSt). No KEGG pathway was found to be associated with the level of Fusobacterium at 20% FDR. The major risk factor for CCA development is chronic biliary inflammation. IL-6 has been shown to promote survival of transformed cholangiocytes through a number of pathways, including the p38 and p44/42 MAPK pathway and through DNA methylation of the promoter region of target genes involved in cancer growth, such as the epidermal growth factor receptor (EGFR) [33].

In recent years, increasing evidence supports the role of the enteric microbiota as a potentially key mediator of liver disease initiation and progression, such as in PSC [34]. Under pathological conditions such as impairment of gut barrier function and modification of gut microbiota composition and potential dysbiosis, the translocation of bacteria or fragments of bacteria from the intestine to the liver can trigger hepatic inflammation and fibrosis [35]. Pathogen-associated molecular patterns (PAMPs) present in the bile can be sensed through the related pattern recognition receptors. The best characterized of these families is the Toll-like receptor (TLR) family. The interaction between lipopolysaccharide (LPS) and TLR4 leads to cholangiocyte release of a broad spectrum of proinflammatory cytokines, such as IL-1β, IL-8, IL-6, MCP-1, TNF-α, IFN-γ and TGF-β [36]. These stimuli enhance liver injury, immune cell infiltration, and induction of hepatic fibrosis. An increasing abundance of oral bacteria in bile of patients with long-term PSC may cause local biliary tract inflammation, may produce biofilm, and may contribute to malignant transformation of the epithelium of the bile duct.

CCA bile had significantly increased abundance of Firmicutes, Fusobacteria, and Actinobacteria compared to control bile. Most CCA in the study were perihilar CCA, since ERCP is much less commonly performed for intrahepatic CCA because palliative biliary stent placement is usually not needed unless patients develop obstructive jaundice. In addition to patients with perihilar CCA, we also had a few cases with intrahepatic or distal cholangiocarcinoma. Our results contrast with two other reports in which the biliary microbiome was compared between patients with bile duct stones and patients with distal CCA: one report mentions that levels of Gemmatimonadetes, Nitrospirae, Chloroflexi, Latescibacteria, and Planctomycetes were significantly higher in CCA patients’ biliary microbiota [9], while the other report describes that Firmicutes, Fusobacteria, and Cyanobacteria levels were significantly lower in bile from CCA cases than patients with bile stones [9]. These differences may reflect regional differences in the biliary microbiota or other yet unrecognized factors.

There are several limitations to our study. Although we included negative controls during DNA extraction, these negative controls do not control for potential contamination during the sampling process. Sampling controls (e.g., air swabs in the surgical suite, swabs of empty tubes or syringes utilized to collect samples) are needed to provide a more rigorous assessment of the degree of contamination in future studies. Given the relatively small sample sizes, the significant results should be interpreted cautiously and need to be replicated in larger studies. Non-significance could be due to type II error (false negative). For example, negative results of predictive functional analysis of the microbiome data may be due to statistical power issues (i.e., small sample sizes, small numbers of disease cases, and high level of noise in the predicted functional profiles by PICRUSt). Therefore, additional large-sample studies as well as shotgun metagenomics are needed to achieve a better understanding of the mechanistic role of Fusobacterium in cholangiocarcinoma pathogenesis.

## 5. Conclusions

Our findings revealed significant changes in the bile microbiome in PSC and CCA. PSC of long duration increased the amount of bile microbiome with an associated increase in specific bile microbiome features, which may increase inflammation in the bile ducts and influence the risk of CCA during the course of PSC. Further detection of specific bile microbial patterns by examination of more subjects may provide additional evidence of critical microbial alterations that could be targeted to develop novel therapies for PSC and alter the risk of CCA in PSC patients. In view of recent observations suggesting that aspirin and statin use are associated with reduced risk of CCA [37,38], as well as evidence that both aspirin and statins alter the stool microbiome, it would also be extremely interesting to investigate the effects of these agents on the bile microbiome.

## Figures and Tables

**Table 1:** Characteristics of study subjects providing bile samples after QC.

Diagnosis (# with Bile 1/# with Bile 2)	PSC (*n* = 28/8)	CCA w PSC (*n* = 21/4)	CCA wo PSC (*n* = 24/7)	Controls (*n* = 5)	*p*
Female, n (%)	10 (36)	3 (14)	6 (25)	3 (60)	NS
Race, white, n (%)	28 (100)	19 (91)	22 (92)	5 (100)	NS
Age (years), Median (IQR)	60 (47–66)	45 (37–52)	62 (51–73)	69 (59–69)	<0.001
BMI (kg/m^2^), Median (IQR)	26 (23–28)	24 (21–29)	27 (22–32)	34 (29–39)	NS
PSC duration (years), Median (IQR)	5 (3–13)	6 (1–10)	NA	NA	NS
CCA site, perihilar, n (%)	NA	20 (95)	23 (96)	NA	NS
Cholelithiasis, n (%)	3 (11)	8 (38)	1 (4)	3 (60)	0.006
Choledocholithiasis, n (%)	3 (11)	0 (0)	4 (17)	4 (80)	<0.001
Leukocytosis, n (%)					
Bile 1	2 (7)	6 (29)	4 (17)	0 (0)	NS
Bile 2	0 (0)	1 (25)	0 (0)	NA	NS
Antibiotic use, n (%)	10 (36)	9 (43)	8 (33)	0 (0)	NS
Use for more than a month	3(11)	4(19)	2(8)	0(0)	NS
MELD score, Median (IQR)	10 (8–14)	18 (10–22)	11 (8–18)	12 (9–12)	NS
Surgical Procedures					NS
Ileal pouch, n (%)	2 (7)	6 (29)	1 (4)	0 (0)	0.038
Ileostomy, n (%)	1 (4)	2 (10)	1 (4)	0 (0)	NS
Cholecystectomy, n (%)	5 (18)	4 (19)	5 (21)	2 (40)	NS
Treatments					
Chemotherapy, n (%)	0 (0)	11 (52)	14 (58)	0 (0)	<0.001
Radiotherapy, n (%)	0 (0)	11 (52)	11 (46)	0 (0)	<0.001
Stent Placement, n (%)	9 (32)	11 (52)	21 (88)	2 (40)	<0.001
Plastic stent *, n (%)	9 (32)	10 (48)	19 (79)	2 (40)	0.008
Metallic stent *, n (%)	0 (0)	1 (5)	5 (21)	0 (0)	0.031
Steroid use (for IBD), n (%)	4 (14)	3 (14)	4 (17)	1 (20)	NS
Immunosuppressant, n (%)	5 (18)	5 (24)	1 (4)	0 (0)	NS
Lifestyle Factors					
Alcohol use, n (%)	14 (50)	11 (52)	15 (63)	2 (40)	NS
Current smoker, n (%)	6 (21)	5 (24)	14 (58)	2 (40)	0.026
Comorbidities					
IBD, n (%)	20 (71)	16 (76)	0 (0)	0 (0)	<0.001
Hypertension, n (%)	15 (54)	5 (24)	14 (58)	3 (60)	NS
Hypercholesterolemia, n (%)	8 (29)	5 (24)	9 (38)	2 (40)	NS
Diabetes mellitus, n (%)	2 (7)	5 (24)	5 (21)	0 (0)	NS

QC, quality control; BMI, body mass index; IBD, inflammatory bowel disease; NA, not available; NS, not significant; *, some patients had both plastic and metallic stents. Comparisons among groups were performed using Chi-square test for categorical variables and Kruskal–Wallis test for continuous variables.

**Table 2:** Characteristics of study subjects providing stool samples after QC.

Diagnosis	PSC (*n* = 31)	CCA w PSC (*n* = 16)	CCA wo PSC (*n* = 11)	*p*
Female, n (%)	12 (39)	5 (31)	3 (27)	NS
Race, white, n (%)	30 (97)	14 (88)	10 (91)	NS
Age (years), Median (IQR)	51 (43–60)	52 (45–55)	60 (51–64)	NS
BMI (kg/m^2^), Median (IQR)	26 (23–28)	25 (23–28)	26 (23–29)	NS
PSC duration (years), Median (IQR)	10 (4–18)	7 (3–12)	NA	NS
CCA site, perihilar, n (%)	NA	12 (75)	9 (82)	NS
Cholelithiasis, n (%)	2 (6)	1 (6)	1 (9)	NS
Choledocholithiasis, n (%)	3 (10)	1 (6)	3 (27)	NS
Leukocytosis, n (%)	2 (7)	5 (31)	0 (0)	0.019
Antibiotic use, n (%)	11 (36)	4 (25)	2 (18)	NS
Use for more than a month	8 (26)	3 (19)	0 (0)	NS
MELD score, Median (IQR)	9 (7–15)	16 (9–22)	11 (8–18)	NS
Surgical Procedures				NS
Ileal pouch, n (%)	8 (26)	5 (31)	2 (18)	NS
Ileostomy, n (%)	5 (16)	2 (14)	2 (18)	NS
Cholecystectomy, n (%)	10 (32)	6 (38)	3 (27)	NS
Treatments				
Chemotherapy, n (%)	0 (0)	6 (38)	2 (18)	0.002
Radiotherapy, n (%)	0 (0)	4 (25)	2 (18)	0.018
Stent Placement, n (%)	3 (10)	5 (31)	9 (82)	<0.001
Plastic stent *, n (%)	3 (10)	5 (31)	7 (64)	0.002
Metallic stent *, n (%)	1 (3)	0 (0)	3 (27)	0.011
Steroid use (for IBD), n (%)	4 (13)	5 (31)	1 (9)	NS
Immunosuppressant, n (%)	3 (10)	2 (13)	1 (9)	NS
Lifestyle Factors				NS
Alcohol use, n (%)	17 (55)	9 (56)	7 (64)	NS
Current smoker, n (%)	1 (3)	4 (25)	3 (27)	0.043
Comorbidities				NS
IBD, n (%)	24 (77)	12 (75)	1 (9)	<0.001
Hypertension, n (%)	8 (26)	3 (19)	4 (36)	NS
Hypercholesterolemia, n (%)	12 (39)	6 (38)	3 (27)	NS
Diabetes mellitus, n (%)	4 (13)	4 (25)	4 (36)	NS

QC, quality control; BMI, body mass index; IBD, inflammatory bowel disease; NA, not available; NS, not significant; *, some patients had both plastic and metallic stents. Comparisons among groups were performed using Chi-square test for categorical variables and Kruskal–Wallis test for continuous variables.

**Table 3:** PERMANOVA *p* values of Demographic and Clinical Factors.

Sample	Bile					Stool				
	UniFrac	WUniFrac	GUniFrac	Bray-Curtis	Omnibus	UniFrac	WUniFrac	GUniFrac	Bray-Curtis	Omnibus
Diagnosis *	0.154	0.414	0.409	0.201	0.347	0.18	0.494	0.339	0.287	0.309
Gender	0.01	0.334	0.107	0.728	0.026	0.869	0.9	0.886	0.814	0.963
Age	0.183	0.154	0.123	0.261	0.291	0.052	0.087	0.075	0.195	0.09
BMI	0.581	0.759	0.866	0.671	0.865	0.509	0.206	0.304	0.507	0.338
IBD	0.555	0.305	0.298	0.046	0.117	0.001	0.035	0.021	0.015	0.001
Cholelithiasis	0.254	0.057	0.053	0.147	0.13	0.903	0.722	0.812	0.344	0.517
Hypertension	0.578	0.24	0.246	0.135	0.312	0.636	0.278	0.261	0.400	0.415
Hypercholesterolemia	0.569	0.81	0.796	0.770	0.861	0.995	0.896	0.928	0.850	0.975
Diabetes mellitus	0.840	0.42	0.629	0.716	0.738	0.455	0.804	0.778	0.708	0.667
Ileal Pouch	0.127	0.015	0.011	0.017	0.029	0.001	0.001	0.001	0.001	0.001
Ileostomy	0.213	0.274	0.279	0.421	0.435	0.001	0.001	0.001	0.001	0.001
Cholecystectomy	0.192	0.031	0.03	0.352	0.076	0.918	0.615	0.704	0.226	0.36
Chemotherapy	0.223	0.043	0.044	0.105	0.116	0.161	0.256	0.132	0.051	0.085
Radiotherapy	0.095	0.36	0.303	0.304	0.231	0.406	0.575	0.429	0.189	0.301
Antibiotics use	0.017	0.165	0.05	0.311	0.045	0.002	0.019	0.005	0.007	0.004
Steroid intake	0.689	0.61	0.596	0.281	0.565	0.074	0.429	0.279	0.374	0.125
Immunosuppressant intake	0.582	0.298	0.332	0.06	0.151	0.278	0.35	0.349	0.574	0.438
Stent placement	0.037	0.18	0.078	0.001	0.001	0.463	0.197	0.289	0.335	0.335

UniFrac, unweighted UniFrac distance; WUniFrac, weighted UniFrac distance; GUniFrac, generalized UniFrac distance; Raw *p* values are presented. *, Diagnosis was a comparison between PSC and CCA wo PSC. Last columns in the Bile and Stool were results of PERMANOVA-based omnibus test [13].

## Data Availability

The data used in this analysis are available with the authors’ permission upon request and ethical approval. The data were uploaded on https://www.ncbi.nlm.nih.gov/bioproject/PRJNA793871, accessed on 19 April 2022.

## Supplementary Materials

The following are available online at https://www.mdpi.com/article/10.3390/cancers14092120/s1, Supplementary file [11,12,13,39,40], Figure S1: Associations between clinical factors and the bile microbiota structure, Figure S2: Associations between clinical factors and the stool microbiota structure, Figure S3: Plots showing microbiome variation, Table S1: Characteristics of study subjects providing bile samples, Table S2, Taxa associated with PSC duration, Table S3: Comparisons of stool microbiome.
