# Supplementary material for: Potential Role of Inflammation-Promoting Biliary Microbiome in Primary Sclerosing Cholangitis and Cholangiocarcinoma
Source: Cancers (Basel). 2022 Apr 24;14(9):2120. doi: 10.3390/cancers14092120 (PMC9104786; doi:10.3390/cancers14092120)
Supplement: Supplementary file 1 [file cancers-14-02120-s001.zip › cancers-1692762-supplementary.pdf]

Potential Role of Inflammation-Promoting Biliary Microbiome in Primary Sclerosing Cholangitis and Cholangiocarcinoma

Katsuyuki Miyabe, Vinay Chandrasekhara, Nicha Wongjarupong, Jun Chen, Lu Yang, Stephen Johnson, Nicholas Chia, Marina Walther-Antonio, Janet Z. Yao, Sean C. Harrington, Cynthia K. Nordyke, John E. Eaton, Andrea A. Gossard, Sharad Oli, Hamdi A. Ali, Sravanthi Lavu, Nasra H. Giama, Fatima A. Hassan, Hawa M. Ali, Felicity T. Enders, Sumera H. Ilyas, Gregory J. Gores, Mark D. Topazian, Purna C. Kashyap, Lewis R. Roberts

Supplementary file

Genomic DNA extraction from stool samples

Fecal DNA was extracted using the PowerSoil kit (MoBio, Carlsbad, CA, USA) according to the manufacturer's instructions and used as a template for the polymerase chain reaction (PCR) targeting 357F and 926R of the bacterial 16S gene. PCR product sizes were verified, and DNA was purified using an epMotion automated system (Eppendorf, Hauppauge, NY) with the Agencourt AMPure PCR Purification System (Beckman Coulter, Brea, CA). Final quantitation was performed using a QuBit HS dsDNA kit and the Qubit Fluorometer 2.0 (Life Technologies, Carlsbad, CA).

Genomic DNA Extraction from Bile Samples using phenol/chloroform extraction

Genomic DNA from bile specimens was obtained by phenol/chloroform extraction and sodium acetate precipitation. Per subject, 1–3 ml of bile was collected and stored at −80°C for DNA extraction. The thawed specimen was transferred to a 2 ml skirted tube, and centrifuged at 18,000 g for 30 min at 4°C. After removing the supernatant, the 0.1mm glass beads were decanted from PowerBead Tubes (QIAGEN 13118-50) into the tube containing the bile pellet, and 500 ul of Buffer A (Tris 191 mM, EDTA 20 mM, NaCl 190.5mM), 210μL of 20% SDS, and 500 ul phenol/chloroform (Phenol/chloroform/Amyl Isopropyl alcohol, Invitrogen 15593-031 pH7.4–8) were added. Homogenization was carried out by bead-beating for 1 minute on the FastPrep 24-G, (setting S = 6, T = 60) twice. After centrifuging the bead tube at 16,000 g for 3min, the aqueous phase was transferred to a new tube for additional phenol/chloroform extraction. After careful transfer of the aqueous phase to a new tube, DNA precipitation was performed by adding 60μL of 3M sodium acetate and 600μL of cold 100% isopropanol and centrifuging at 16,000 g at 4°C for 20 minutes. The DNA pellet was then washed in cold 100% ethanol. After dissolving the DNA pellet in PBS, a clean-up procedure was performed to remove the phenol/chloroform residue from the DNA samples using the QIAGEN DNeasy Blood & Tissue Kit (QIAGEN 69504). To determine DNA concentration, the Qubit dsDNA assay was used and the results read using the Qubit Fluorometer 2.0.

A pooled human stool aliquot (250 mg+/−) and a blank sample tube were processed alongside the bile samples as positive and negative controls, respectively.

Next generation sequencing and bioinformatics processing of the sequenced reads

Both bile and stool 16S rDNA amplicons were pooled to equal concentration and sequenced on one lane of a MiSeq at the Mayo Genomics Facility using the MiSeq Reagent Kit v2 (2 × 250 reads, 500 cycles; Illumina Inc., San Diego, CA). Pre-processed sequence files were then processed via the *hybrid-denovo* bioinformatics pipeline [1] with the default parameter setting to form operational taxonomic units (OTUs). *Hybrid-denovo* improves over IM-TORNADO[1] by using a mixture of single-end and paired-end reads to form OTUs, and was shown to be more sensitive than methods using either single-end or paired-end reads. OTUs were assigned taxonomy using the RDP classifier trained on the Greengenes database (v13.5) and a phylogenetic tree was built based on FastTree [3].

Statistical analyses

Differences of patients' characteristics among disease groups were compared using the Chi-square test or Fisher's exact test for categorical variables and the Kruskal-Wallis test for continuous variables.

Statistical analysis of microbiome was performed in two steps. First, sources of variability in the dataset were identified by testing associations between the overall microbiota composition and various clinical variables using the PERMANOVA-based omnibus test combining UniFrac, GUniFrac ($\alpha = 0.5$), Weighted UniFrac and Bray-Curtis distances ("PermanovaG" in R "GUniFrac" package v1.1) [4]. Separate tests were conducted for the bile and stool samples; for bile samples, only one bile sample was used for each case to satisfy the independence assumption. The results were adjusted for clinical variables that were significantly associated with the bile and stool microbiota with a p-value < 0.05.

Next, detailed comparative analyses of the bile and stool microbiota from PSC and CCA patients were conducted. Analyses were performed at α-diversity, β-diversity and taxa abundances. α-diversity reflects species richness and

evenness within the microbial community. Two α-diversity indices were calculated: observed number of OTUs (species richness measure) and Shannon index (overall diversity measure) on the rarefied data. A linear regression model was used for testing the association with α-diversity adjusting for potential confounders. β-diversity reflects the shared diversity between microbial communities in terms of ecological distance; pairwise distance metrics allow quantification of differences between samples. Four β-diversity measures (unweighted UniFrac distance, weighted UniFrac distance, generalized UniFrac distance (α = 0.5), and Bray-Curtis distance) were calculated (R packages "GUniFrac" v1.1 and "vegan" v2.4.3) on the rarefied data. To test the association with β-diversity measures, we used the PERMANOVA-based omnibus test (999 permutations). This was the same procedure used for identifying sources of variability.

Taxa-level associations were performed at the phylum, class, order, family, genus and OTU levels, and taxa with prevalences less than 10% or with a maximum proportion less than 0.2% were excluded from testing to reduce the number of the tests. The count data was normalized into relative abundances by dividing by the GMPR size factor to address potential compositional effects [5]. To identify differentially abundant taxa associated with the variable of interest while adjusting for potential confounders, a permutation-based approach (999 permutations) was used with the F-statistic of a linear model (square-root transformed taxa relative abundance as the response variable) as the test statistic. An FDR control (BH procedure, 'p.adjust' in R 3.3.2) was used to correct for multiple testing at each taxonomic level. For the main analyses, we used 10% FDR to select taxa. For some scenarios, when there was evidence of association at the community level analysis but the individual associations were weak, we relaxed the FDR level to 20%.

To test whether the bile and stool sample from the same subject were similar, a distance-based permutation approach was used. Specifically, the average distance between the bile and stool samples from the same subjects (Dw) was compared to the average distance between the bile and stool samples from different subjects (Db). If Db > Dw, it indicated the bile and stool sample from the same subject were more similar. The test statistic Ts = (Db − Dw) ("similarity index") was calculated as the test statistic. Permutation was then used to assess significance, and the reported p-value was the percentage of Ts under permutation larger than the observed Ts. To identify specific taxa driving the overall similarity, we defined a taxon-specific distance based on Euclidean distance on square-root transformed relative abundances. Similar permutation tests were used. Statistical analyses were performed in R 3.3.2 and JMP software (version 10.0.2; SAS Institute, Cary, NC, USA).

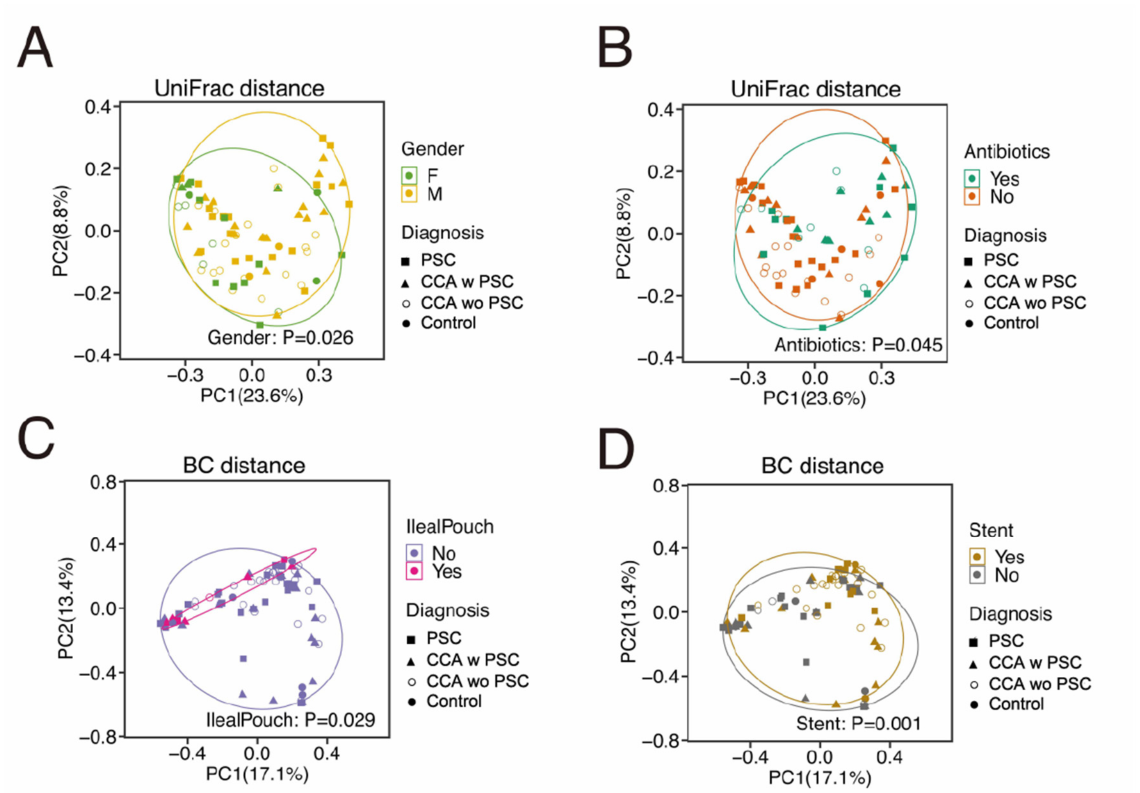

Figure S1. Associations between clinical factors and the bile microbiota structure. PCoA plots showing effects of gender (A), antibiotics (B), presence of an ileal pouch (C) and stent placement (D).

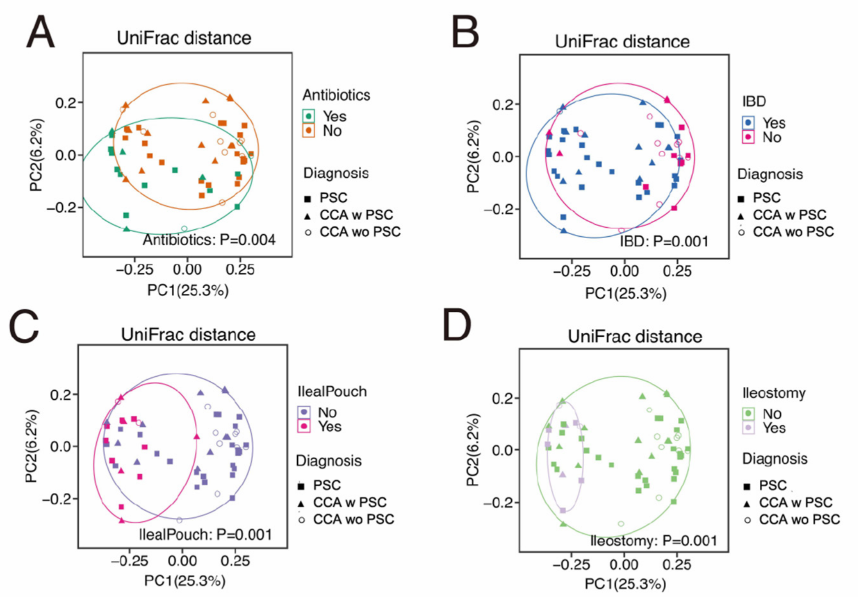

Figure S2. Associations between clinical factors and the stool microbiota structure. Principal coordinate analysis (PCoA) plots showing effects of antibiotics (A), coexisting IBD (B), presence of an ileal pouch (C), and presence of an ileostomy (D).

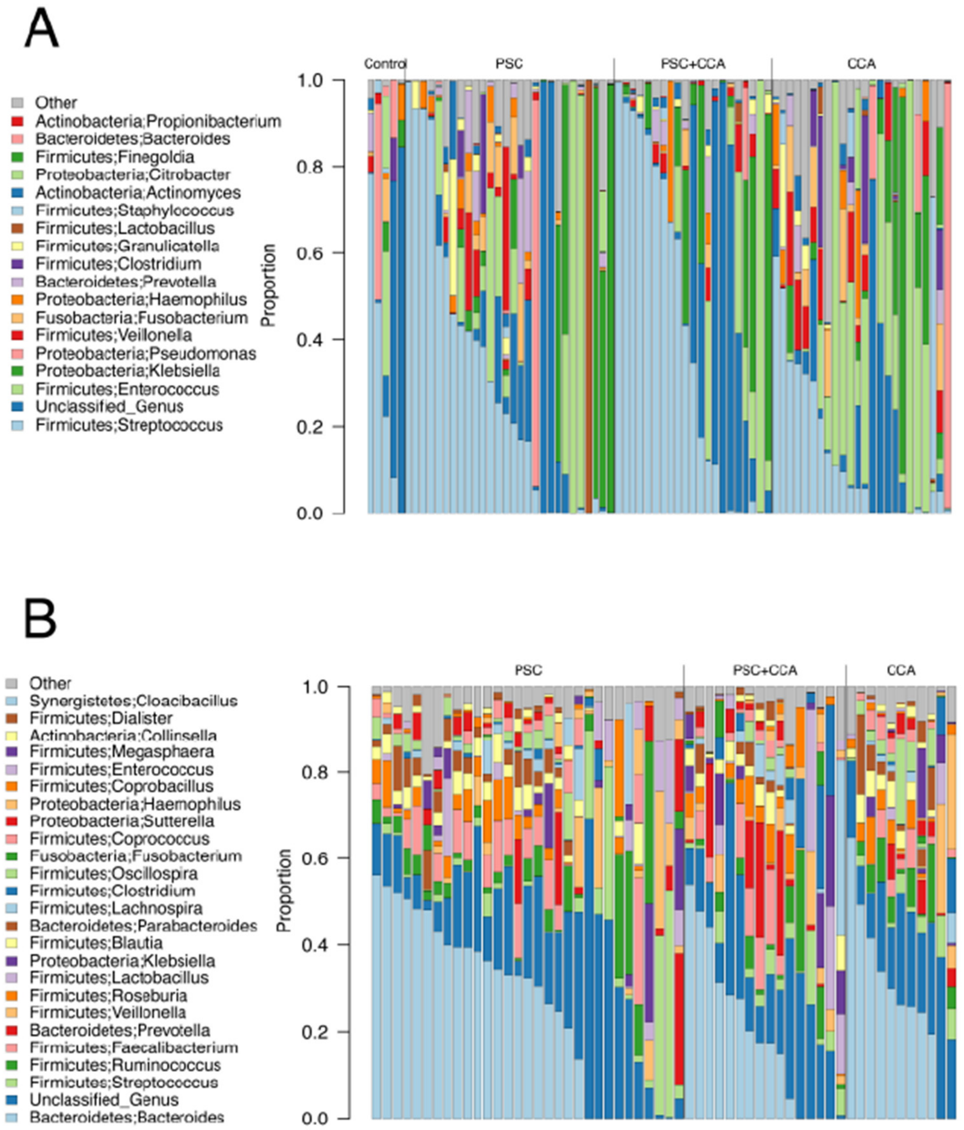

Figure S3. Plots showing microbiome variation in bile (A) and stool (B) from patients with PSC, PSC associated CCA, CCA wo PSC, and controls (for bile only).

Table S1. Characteristics of Study Subjects Providing Bile Samples.

**Diagnosis (# with Bile 1/# with Bile 2)**	**PSC (*n* = 32/9)**	**CCA w PSC (*n* = 23/7)**	**CCA wo PSC (*n* = 26/7)**	**Controls (*n* = 17/0)**	***p***
Female, n (%)	11 (34)	5 (22)	8 (31)	9 (53)	NS
Race, white, n (%)	28 (88)	19 (83)	22 (85)	15 (88)	NS
Age (years), Median (IQR)	59 (44-65)	45 (38-52)	62 (49-72)	55 (42-69)	0.001
BMI (kg/m^2), Median (IQR)	26 (23-28)	24 (21-28)	27 (22-33)	30 (27-33)	0.032
PSC duration (years), Median (IQR)	5 (2-11)	6 (0-10)	NA	NA	NS
CCA site, perihilar, n (%)	NA	21 (91)	25 (96)	NA	NS
Cholelithiasis, n (%)	3 (9)	2 (9)	8 (31)	15 (88)	0.001
Choledocholithiasis, n (%)	3 (9)	1 (14)	4 (15)	7 (41)	0.016
Leukocytosis, n (%)					
Bile 1	2 (6)	7 (30)	4 (15)	0 (0)	0.001
Bile 2	0(0)	1(4)	0 (0)	NA	NS
Antibiotic use, n (%)	11 (34)	9 (39)	8 (31)	1 (6)	NS
Use for more than a month	3 (9)	4 (19)	2 (8)	0 (0)	NS
MELD score, Median (IQR)	10 (8-13)	16 (11-22)	11 (8-19)	9 (8-11)	NS
Surgical Procedures					
Ileal pouch, n (%)	2 (6)	6 (26)	1 (4)	0 (0)	0.016
Ileostomy, n (%)	1 (3)	2 (9)	1 (4)	0 (0)	NS
Cholecystectomy, n (%)	6 (19)	4 (17)	5 (19)	2 (12)	NS
Treatments					
Chemotherapy, n (%)	0 (0)	11 (48)	14 (54)	0 (0)	<0.001
Radiotherapy, n (%)	0 (0)	11 (48)	11 (42)	0 (0)	<0.001
Stent Placement, n (%)	9 (28)	11 (48)	22 (85)	2 (12)	<0.001
Plastic stent *, n (%)	9 (28)	10 (43)	20 (77)	2 (12)	<0.001
Metallic stent *, n (%)	0 (0)	1 (4)	5 (19)	0 (0)	0.010
Steroid use (for IBD), n (%)	4 (13)	3 (13)	4 (15)	1 (6)	NS
Immunosuppressant, n (%)	5 (16)	5 (22)	1 (4)	0 (0)	0.036
Lifestyle Factors					
Alcohol use, n (%)	17 (53)	12 (52)	17 (65)	7 (41)	NS
Current smoker, n (%)	7 (22)	5 (22)	14 (54)	8 (47)	0.024
Comorbidities					
IBD, n (%)	24 (75)	16 (70)	0 (0)	1 (6)	<0.001
Hypertension, n (%)	17 (53)	5 (22)	14 (54)	11 (65)	0.024
Hypercholesterolemia, n (%)	10 (31)	6 (26)	9 (35)	4 (24)	NS
Diabetes mellitus, n (%)	2 (6)	5 (22)	6 (23)	1(6)	NS

QC, quality control; BMI, body mass index; IBD, inflammatory bowel disease; NA, not available; NS, not significant; *, some patients had both plastic and metallic stents. Comparisons among groups were performed using Chi-square test for categorical variables and Kruskal-Wallis test for continuous variables.

Table S2. Taxa Associated with PSC Duration (FDR<20%).

	***p* value**	***q* value**	**R2**	**Coefficient**	**Spearman's Rank-Order Correlation**
Firmicutes; Gemellales	0.006	0.108	0.18	0.002	0.394
OTU194: Firmicutes; Megasphaera	0.01	0.169	0.162	0.001	0.437
OTU166: Firmicutes; Gemella	0.007	0.138	0.168	0.001	0.398
OTU213: Actinobacteria; Actinomyces	0.007	0.138	0.151	0.001	0.357
OTU280: Bacteroidetes; Prevotella	0.003	0.118	0.23	0.002	0.454
OTU112: Firmicutes; Veillonella	0.001	0.059	0.251	0.002	0.46
OTU152: Bacteroidetes; Prevotella	0.005	0.138	0.182	0.001	0.47
OTU1290: Fusobacteria; Fusobacterium	0.001	0.059	0.266	0.001	0.336

Table S3. Comparisons of stool microbiome.

	Observed OTUs	Shannon index	Unweighted UniFrac	Weighted UniFrac	Generalized UniFrac	Bray-Curtis	Omnibus
PSC vs. CCA w PSC	0.28	0.41	0.42	0.36	0.51	0.25	0.38
CCA w PSC vs. CCA wo PSC	0.59	0.66	0.42	0.66	0.67	0.78	0.62

A linear regression model was used for testing the association with α -diversity adjusting for potential confounders. To test the association with β-diversity measures, we used the PERMANOVA-based omnibus test (see **Supplementary material**).

References

1. Chen, X., Johnson, S., Jeraldo, P., Wang, J., Chia, N., Kocher, J.A., Chen, J. Hybrid-denovo: a de novo OTU-picking pipeline integrating single-end and paired-end 16S sequence tags. *Gigascience*. **2018**, *7*, 1–7.
2. Jeraldo, P., Kalari, K., Chen, X., Bhavsar, J., Mangalam, A., White, B., Nelson, H., Kocher, J., Chia, N. IM-TORNADO: a tool for comparison of 16S reads from paired-end libraries. *PLoS One*, **2014**, *9*, e114804.
3. Price, M.N., Dehal, P.S., Arkin, A.P. FastTree 2—approximately maximum-likelihood trees for large alignments. *PLoS One*, **2010**, *5*, e9490.
4. Chen, J., Bittinger, K., Charlson, E. S., Hoffmann, C., Lewis, J., Wu, G. D., Collman, R. G., Bushman, F. D., & Li, H. Associating microbiome composition with environmental covariates using generalized UniFrac distances. *Bioinformatics*. **2012**, *28*, 2106–2113.
5. Chen, L., Reeve, J., Zhang, L., Huang, S., Wang, X., Chen, J.. GMPR: A robust normalization method for zero-inflated count data with application to microbiome sequencing data. *PeerJ*. **2018**, *6*, e4600.
